# Detecting Pre-Frailty or Frailty Using Activity Monitoring from Wearable Sensor

**DOI:** 10.1093/geroni/igaf122.4315

**Published:** 2025-12-31

**Authors:** Andrew Song, Kailin Xu, Kuan-Yuan Wang, Lingsong Kong, Dae Hyun Kim

**Affiliations:** Hebrew Seniorlife, Roslindale, Massachusetts, United States; Hebrew Seniorlife, Roslindale, Massachusetts, United States; Hebrew SeniorLife, Roslindale, Massachusetts, United States; University of Massachusetts, Amherst, Massachusetts, United States; Hebrew SeniorLife, Boston, Massachusetts, United States

## Abstract

Early identification of pre-frailty or frailty in community-dwelling older adults can enable targeted interventions, yet clinical assessments are time-consuming and rarely used in routine care. We evaluated whether activPAL, a thigh-worn accelerometer, can identify pre-frail-or-frail status in older adults. In a cross-sectional study, we enrolled 44 participants aged 63–97 years from independent living or assisted living facilities within Hebrew SeniorLife. They completed the Fried Frailty Phenotype (FFP) and Comprehensive Geriatric Assessment-based Frailty Index (CGA-FI) assessments, then wore activPAL for consecutive 10 days. We derived 38 features across activity volume, gait, sedentary behavior, and diurnal patterns from the activPAL data. Lasso and ridge regression, random forest, and a simple neural network were trained to classify robust versus pre-frail-or-frail against each frailty measure. Performance was evaluated using area under the ROC curve, balanced accuracy, and F1 score with nested cross validation. 36 participants were pre-frail-or-frail by FFP (≥1 point) and 28 by CGA-FI (≥0.15). The pre-frail-or-frail group showed less walking time, lower cadence, smaller activity amplitude, and lower variability in total sedentary time. Ridge regression performed better than other models for predicting pre-frail-or-frail by FFP (AUC, 0.81; balanced accuracy, 0.65; F1, 0.76) and by CGA-FI (AUC, 0.78; balanced accuracy, 0.71; F1, 0.79). Our findings demonstrate that thigh-worn activity monitoring can be useful in detecting pre-frail and frail status, highlighting its potential as a scalable, sensor-based screening tool to guide early preventive interventions in older adults. External validation in a larger, diverse sample is warranted.

